# School-based cardiopulmonary resuscitation training for high school students in Brazil: a pre-post educational intervention with 3-month follow-up

**DOI:** 10.1016/j.bjane.2026.844787

**Published:** 2026-07-01

**Authors:** Felipe Batista Santos, Leonardo Santana Ramos Oliveira, Julio Cezar de Oliveira Filho, Fabricio Dias Antunes

**Affiliations:** a Universidade Federal de Sergipe, Aracaju, SE, Brazil; b Universidade Federal de Sergipe, Residency Program, Aracaju, SE, Brazil

**Keywords:** Basic life support, Cardiopulmonary resuscitation, Health education, Heart arrest, Schools, Students

## Abstract

**Background:**

Cardiac Arrest (CA) is considered a medical emergency and survival after CA is approximately 10%. Recognizing and providing effective intervention can have a significant impact on a patient's outcome.

**Objective:**

To evaluate baseline knowledge of Cardiopulmonary Resuscitation (CPR) and the impact of a CPR training intervention to high school students in Aracaju, Sergipe.

**Methods:**

This study was a pre–post educational intervention with 3-month follow-up involving 5 institutions that were chosen randomly from March to October 2023. The intervention was delivered in Portuguese, in four stages, aligned with the Kids Save Lives initiative: a pre-test was applied in 410 students before starting the course with questions regarding CPR and students’ expectations; a theoretical-practical course was taught; after the course, students answered a post-test, an evaluation and satisfaction questionnaire. Finally, three months later, 343 students answered the post-test.

**Results:**

The primary outcome was mean total score on a validated 14-item BLS knowledge test. We observed an increase in scores after training from 5.8 (2.2) pre-test to 10.9 (2.0) three months after. Scores decreased significantly from the immediate post-test to the 3-month follow-up, with a mean difference of 0.9 points (95% CI 0.61 to 1.13; Cohen’s *d* = 0.354), but remained substantially higher than baseline. Self-perceived ability to perform CPR increased from 10.9% before training to 91% immediately after the course.

**Conclusion:**

Teaching CPR in educational institutions can improve CPR knowledge retention at 3 months following the intervention, supporting the feasibility of integrating similar programs in Brazilian schools and alignment with the trend of Brazilian education policies.

**Institutional Research Board approval:**

CAAE number: 57353622.0.0000.5546.

## Introduction

The American Heart Association (AHA) defines Cardiac Arrest (CA) as the sudden complete loss of cardiac function in an individual, regardless of whether they have a history of heart disease, and symptoms being present or not beforehand.^1^ The overall survival rate in out-of-hospital CA cases is currently around 10%, and this rate is directly related to the circumstances in which the event occurs, such as whether it happens at home, whether it is witnessed, and whether emergency services are accessible nearby, among other factors.^1^, ^2^, ^3^ Prompt recognition and the provision of swift and effective initial intervention can have a significant impact on the patient’s clinical outcome.^4^ This involves following the ongoing guidelines provided by AHA, an organization that, among its various initiatives, promotes the dissemination of Basic Life Support (BLS) practices to the general public.

In 2015, the World Health Organization (WHO) endorsed the Kids Save Lives (KSL) project, which was launched in 2012 in Italy and Germany. The objective of this project is to introduce the teaching of CPR in schools in order to spread knowledge and improve social indicators related to out-of-hospital CA deaths.^5^, ^6^, ^7^, ^8^ In Brazil, the initiative began to be implemented by USP (Universidade de São Paulo) and spread to other states. However, there is still no record of its application in Sergipe.

The primary objective was to evaluate the effect of a structured CPR training intervention in Portuguese on high school students’ knowledge using a 14-item test, measured before, immediately after, and at 3 months post-training. This initiative was conducted within the public-school network of Aracaju and aligned with the national Kids Save Lives Brazil (“KSLB”) efforts (https://sites.usp.br/kidssavelivesbrasil/).^9^, ^10^, ^11^

## Methods

This is an exploratory, descriptive, and longitudinal study with a quantitative approach based on data obtained through questionnaires. The study involved 343 students from five randomly selected public and private educational institutions, conducted from March to October 2023. The training consisted of theoretical and practical courses with an average duration of three hours, focusing on the topic of BLS. The theoretical portion was delivered through a lecture, followed by a practical session using manikins to demonstrate the BLS procedures.

The methodology of this study, as well as its ethical aspects, was evaluated by the Research Ethics Committee of the University Hospital of the Federal University of Sergipe, which issued an approval opinion under CAAE number 57353622.0.0000.5546. Written informed consent was obtained from all participants and/or their guardians as per local requirements, in Portuguese.

The study included high school students of any gender from public and private schools. The exclusion criteria were incomplete questionnaire responses and failure to complete the training course.

The study was divided into four stages: the first consisted of a pre-test (objective theoretical questions) and an expectations questionnaire before the course, that was applied to 410 students; the second was the theoretical-practical course; in the third, immediately after the course, students completed a post-test identical to the pre-test and a satisfaction/evaluation questionnaire; and finally, in the fourth stage, a follow-up post-test was administered three months after the course, to 343 students.

The data collection instrument used to assess technical knowledge in BLS (pre-test, immediate post-test, and delayed post-test) comprised 14 questions, each worth one point, totaling a maximum score of 14. Questions 1 to 3 addressed initial CPR measures; questions 4 and 5 addressed CA recognition; questions 6 to 9 focused on seeking help; and questions 10 to 14 covered CPR techniques. The full list of the items used to assess understanding is provided in Supplementary Data. Content validation was conducted by a panel of CPR experts, and the questions were based on international guidelines and KSL initiatives.

The expectation and satisfaction/evaluation questionnaires included variables using the Likert scale format, with some questions expressed numerically and others categorized as: strongly agree, agree, neutral, disagree, and strongly disagree.

The sample size was estimated using a two-sided α = 0.05, power = 0.80, and assumed a medium effect size (Cohen’s *d* = 0.50). Sample size was adjusted for clustering using a design effect calculated with an Intraclass Correlation Coefficient (ICC) of 0.05 and an average cluster size of 80 students. An additional 30% inflation was applied to account for anticipated loss to follow-up. The final required sample size was 223 students. However, although an a priori sample size calculation was estimated, the final sample should be interpreted as a convenience sample based on feasibility and the availability of participating schools.

Categorical variables were analyzed by determining the absolute and relative frequencies of the responses, while numerical variables were characterized using means and standard deviations. The variation in scores between the pre- and post-tests was analyzed using repeated measures analysis of variance (ANOVA), considering the influence of the educational institutions. The effect of time on mean scores between the pre-test, immediate post-test, and delayed post-test (three months later) was evaluated using repeated measures ANOVA. To control for the effects of the different schools (since the educational activities occurred on different days and locations), the model was adjusted accordingly considering school as a random effect to account for the clustering of students within educational. A significance level of 5% was adopted to determine the statistical importance of the results. Statistical analysis was performed using R software, version 4.2.3.

## Results

The study included 343 high school students from five schools in a Brazilian municipality, comprising four private institutions and one public institution. The description of the participants is presented in Table 1. Initially, 410 students were enrolled in the study, 410 completed the pre-test, 410 the immediate post-test, and 343 the 3-month follow-up. The main reasons for this reduction in sample size were participant absence during data collection and difficulties in establishing contact or securing follow-up for some individuals. All statistical analyses were therefore performed using only the final group of students who completed the entire study (n = 343).

**Table 1:** Description of the individuals included.

Variable	n = 343^a^
Gender	
Female	181 (52.8%)
Male	160 (46.6%)
Not informed	2 (0.6%)
Educational Institution	
A	49 (14.3%)
B	41 (12%)
C	70 (20.4%)
D	121 (35.3%)
E	62 (18%)
School Year	
First Year	30 (8.7%)
Second Year	119 (34.7%)
Third Year	194 (56.6%)
Age (years)^b^	17 (16-18)

a n (%).

b Median (IQR).

Most participants were female (52.8%), with 46.6% being male students, and only 0.6% chose not to disclose their gender. Additionally, 56.6% of the participants were enrolled in the third year of high school. It is important to note that this study did not differentiate between private and public institutions.

**Figure 1:**
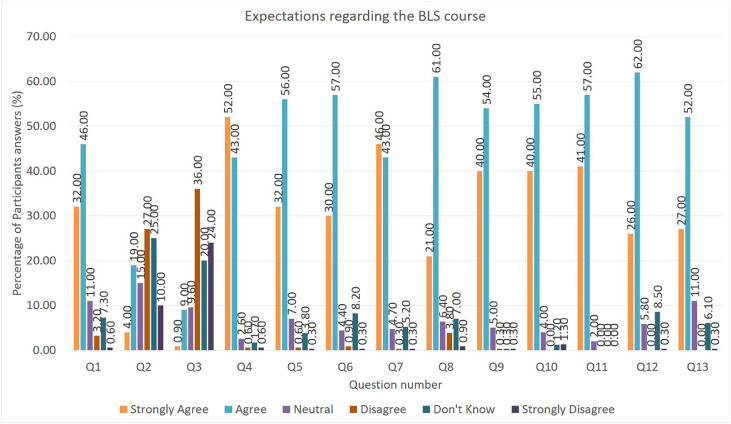
Expectations regarding the BLS course.

In Figure 1, shown below, data relate to the participants' expectations regarding the CPR course, in the BLS training.

The enthusiastic participation of 78% of those involved in the CPR course is a notable highlight, contrasting with the small percentage of 3.8% who appeared less motivated. Additionally, 7.3% remained neutral regarding their motivation. Regarding current knowledge, 39.4% felt prepared, 37% expressed uncertainty, and 25% chose not to state an opinion. When it came to practical skills, 10.9% felt confident, while 60% revealed lack of confidence, and 20% were undecided.

The will to learn was evident, with 95% eager to expand their knowledge during the course. This outlook was shared by the vast majority (88%) who hoped to master resuscitation techniques, and by 87% who were confident in their ability to assimilate the proposed content. The simulation with manikins was considered essential by 89% of the participants.

The quality of the resources and activities offered in the course was also a point of confidence, with an impressive 82% of participants believing it would be sufficient for their learning. Furthermore, the majority (94%) expected to rely on the indispensable support of the tutors for good performance, and 95% were confident that the instructors would be accessible to clarify any doubts that might arise.

The analysis of the participants' score data was conducted using ANOVA, as it provided better interpretation of the results and allowed for the calculation of effect size. ANOVA showed an intervention effect on participants' scores (FGreenhouse-Geisser [1.95; 657.83] = 998.4, p < 0.001, η²p = 0.747). Scheffé’s post hoc test indicated a statistical difference between the means of all three points in time (p < 0.001), with the pre-test mean of 5.8 with a standard deviation of 2.2; the immediate post-test mean was 11.8 with a standard deviation of 1.6; and the post-test conducted three months after the course showed a mean of 10.9 with a standard deviation of 2.0. Mean score three months later showed a significant difference compared to the immediate post-test (95% CI 0.61 to 1.13, Cohen’s *d* = 0.354).

**Figure 2:**
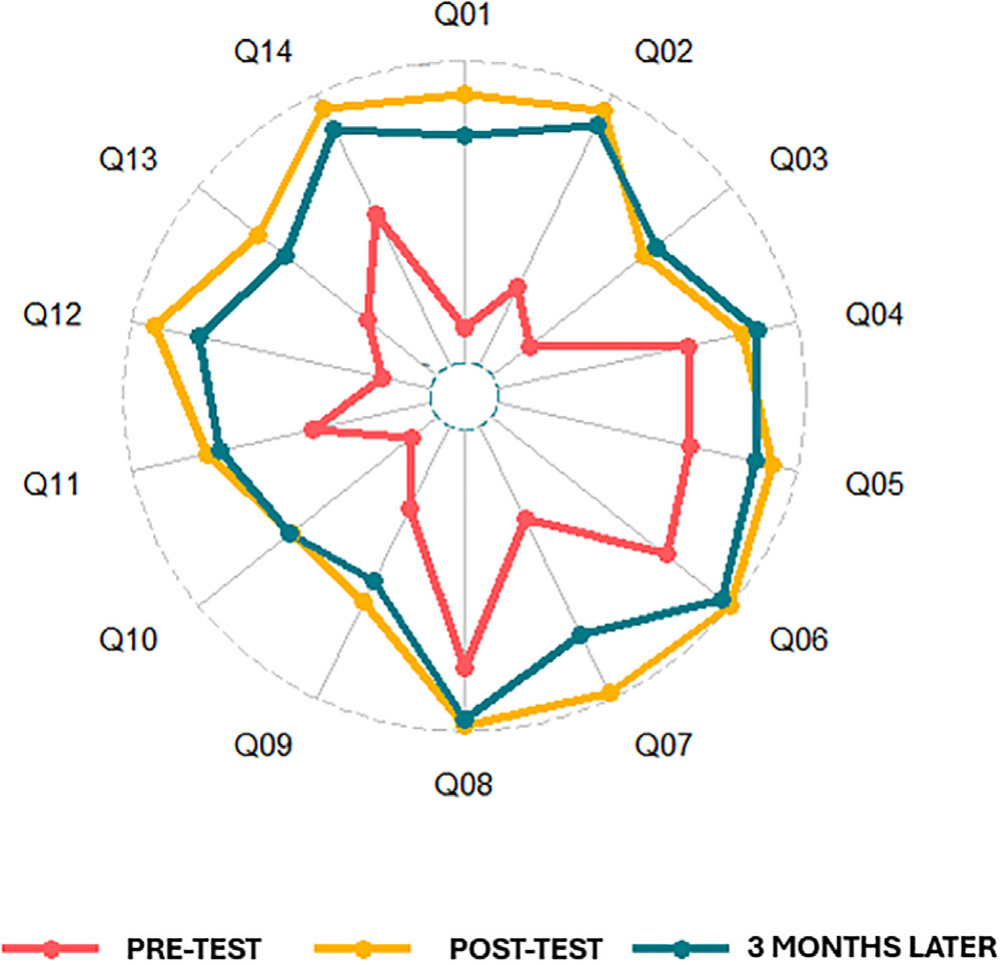
Pattern of correct answers in the pre-test, immediate post-test, and 3-month post-test.

The pattern of correct answers by the participants is shown in Figure 2. After the educational intervention, a notable improvement was observed in participants’ correct responses. Furthermore, it is evident that individuals had significantly higher prior knowledge regarding seeking help ‒ knowing which number to call for emergency services (SAMU) and where to find an Automated External Defibrillator (AED) ‒ compared to their understanding of the subsequent steps in managing CA. On the other hand, in the delayed post-test, a correct answer rate of approximately 60% was observed for questions 9, 10, and 13, indicating a possible decline in the retention of knowledge related to the steps to be followed during CA management when compared to performance on the rest of the test.

**Figure 3:**
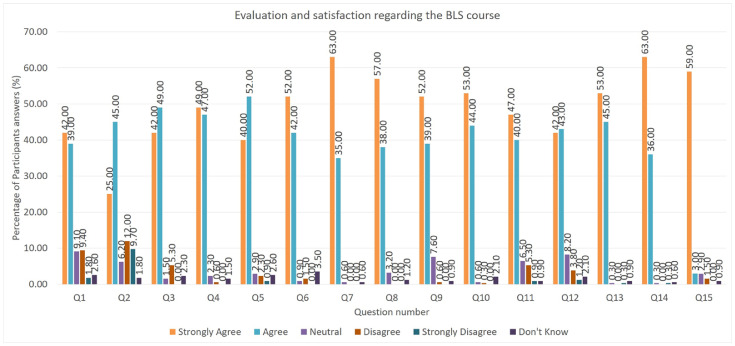
Evaluation and satisfaction regarding the BLS course.

Figure 3 describes the participants’ evaluation and satisfaction regarding the activity after the course. In this questionnaire, emphasis is placed on the combined percentage of those who completely agree and those who simply agree with the statements. When comparing these results to the expectation questionnaire (Fig. 1), it is noted that the participants’ motivation remained virtually the same (77%). After the intervention, 70% of students perceived their knowledge as sufficient, 91% regarding the ease of performing CPR maneuvers after the practical class, 92% regarding the ease of executing the step-by-step process to manage a CA after both theoretical and practical classes, and 98% regarding the importance of the manikin simulation in consolidating theoretical knowledge. The students also evaluated the overall organization of the course, as illustrated in Table 2.

**Table 2:** Results of evaluation of the BLS course structure.

Statement	Mean (SD)	Median [IQR]	Minimum ‒ Maximum
General organization and course structure	9.3 (0.91)	10 [9 ‒ 10]	**5 ‒ 10**
Teaching staff and tutors (different skills, actions and strategies adopted by teachers and tutors in the development and promotion of the course)	9.4 (0.95)	10 [9 ‒ 10]	**3 ‒ 10**
Assessment system (relevance of theoretical assessment methods for student learning)	**9.3 (1.16)**	**10 [8 – 10]**	**5 ‒ 10**
Materials provided (quality, interest and applicability of materials provided in the course)	9.4 (0.91)	10 [9 ‒ 10]	**5 ‒ 10**
Work methodologies (how the content is worked on in group dynamics)	9.4 (0.91)	10 [9 ‒ 10]	**5 ‒ 10**
Support services (ways of supporting students throughout the course in terms of resolving queries and relating to learning progression)	9.6 (0.81)	10 [9 ‒ 10]	**5 ‒ 10**

## Discussion

This work represents a local implementation and replication of school-based CPR training, aligned with ongoing Brazilian initiatives.^2^^,^^10^ Our findings demonstrate consistent knowledge retention at 3 months, complementing prior Kids Save Lives Brazil programs.^2^^,^^10^ The study’s novelty is in adapting, delivering, and reporting outcomes within a specific school network (Aracaju, Sergipe).

The low cost of implementing the CPR program in schools stands out due to the simplicity of the materials required, such as BLS manikins and educational videos. With volunteer instructors, such as local healthcare professionals, conducting the training sessions, costs are further reduced.

The schools selected for this study were strategically chosen to represent the social and educational diversity of the capital city. In the context of this study, the implementation of a 180-minute instructional program, which included the use of intermediate-fidelity manikins in schools, proved to have a significant impact, resulting in substantial improvements in evaluations between the pre-test, immediate post-test, and delayed post-test.^11^

Most students, around 70% of participants, had prior knowledge about the need to seek help in the event of CA. However, only about 15% of students knew how to carry out the initial steps required after recognizing CA. These findings are consistent with the results of a study conducted with second-year high school students in Ceará, which revealed a low accuracy rate in the pre-test regarding the assessment of responsiveness and CPR technique.^11^

The analysis of the results from the present study indicates that the initial intervention was effective in improving participants’ knowledge about CPR, as the average score in the immediate post-test was 11.8, corresponding to an 84.3% success rate, while the average score in the pre-test was 5.8, equivalent to about 41.4% accuracy. Similar results were observed in studies conducted with high school students in Saudi Arabia, Jordan, and Brazil. Initially, these studies identified low prior knowledge among students regarding the management of CA. However, following educational interventions, participants demonstrated a significant increase in acquired knowledge and skills.^12^, ^13^, ^14^

It is worth noting that the results show a slight decrease in the accuracy rates of the questions in the delayed post-test compared to the immediate post-test. However, scores remained substantially higher than baseline despite the small decline over time, indicating a likely retention of learning over the three-month period. When analyzing the post-test question by question, questions 9, 10, and 13 from the test (Fig. 2) showed an accuracy rate of around 60% in the delayed post-test, indicating lower performance compared to the other questions in the same test. However, the outcome for these questions was nearly the same in the immediate post-test. This suggests that more technical questions may present a higher level of difficulty for lay responders. Similarly, an evaluation of a study conducted with second-year high school students in the city of Maceió/AL showed a significant reduction in the percentage of correct answers in the delayed evaluation when the topic was related to the technical aspects of chest compression maneuvers.^11^ In this context, considering the nature of the specific questions on the post-test is important, as some questions maintained high accuracy rates, reaching up to 96.5%, possibly due to their association with easily memorized information.

When compared with the existing literature, the results of this study are consistent with previous research showing that CPR knowledge retention can decline over time, even if gradually, following the successful completion of an initial training course.^11^ This highlights the importance of continuing education and regular practice to maintain CPR skills. In any case, when comparing the results of the delayed post-test with the pre-test, a considerable improvement in learning can still be observed; however, a study conducted over a longer analysis period is indeed necessary. It is worth noting that several authors have studied this topic, but there is no consensus in the literature regarding the ideal interval between training sessions, which may vary depending on several parameters.^14^ Essential skills, like calling for assistance, performing chest compressions, and providing ventilation, tend to deteriorate between three and six months after the initial training. It is important to note that these findings are based on various studies that differ in terms of course duration and format, the characteristics of the instructors and participants, as well as the frequency with which participants are involved in real-life resuscitation situations.^14^, ^15^, ^21^ As an example, Berden et al.^15^ concluded that for a group of nurses in non-cardiac units, satisfactory maintenance of CPR skills could be achieved with training every six months. However, Woollard et al.,^16^ when training lay individuals at an airport in the United Kingdom, suggested that the interval between CPR and AED training sessions should not exceed seven months. On the other hand, Riegel et al.,^17^ when studying lay volunteers, found an adequate level of retention of CPR and AED skills even 17 months after the initial training.

After completing the course, the vast majority of participants reported an increase in their motivation (77%), knowledge about CPR (96%), and confidence in performing CPR maneuvers (91%). It is worth noting that 98% of participants considered the manikin simulation during the practical activity to be essential for consolidating CPR content. Furthermore, most students (60%) stated, prior to the course, that they did not feel confident in managing CA following the CPR protocol. After the course, there was a significant increase in that confidence to 92%. The data obtained in this study support the positive impact of the course and reinforce findings from several studies in the literature.^18^^,^^19^

Students gave positive evaluations, with average scores ranging from 9.3 to 9.6, for the overall organization of the course, support services, teaching methodology, and materials provided. These results reflect the importance and the recommendation given by the participants, with 98% stating they would recommend the course to a friend or family member, and 99% considering the course important for society in general. Additionally, 94% of participants agreed that the course should be mandatory in all schools. In this regard, redirecting educational efforts toward the youth population in schools represents an effective strategy to reach a broad social spectrum, given the unique capacity of this environment to bring together a large number of people with the shared goal of learning.^2^^,^^6^ The main objective of the KSL initiative is to provide BLS education to children of all ages, with a special focus on those over 12 years-old, who generally possess the physical ability required to perform the full BLS protocol.^12^ However, even younger children (up to 5 years-old) can be taught CPR concepts, with an emphasis on spreading knowledge or guiding adults on how to perform CPR during a CA, including the use of an automated external defibrillator. Studies have shown that younger children, after repeated exposure to the topic, are capable of retaining and transmitting the information appropriately, effectively playing the role of rescuers or instructors for others.^9^^,^^20^ Continuous training promotes an increase in confidence and reaction speed, resulting in a significant increase, by 3 to 5 times, in the chances of survival for out-of-hospital CA victims, especially when the event is witnessed.^1^^,^^6^^,^^7^^,^^9^

This study has some limitations that should be acknowledged. First, the sample was limited to five educational institutions in a single Brazilian municipality, which limits the interpretation of the findings to other regions with different socioeconomic and cultural characteristics. Second, the relatively short follow-up period (3 months) does not allow for assessing long-term knowledge and skill retention beyond this timeframe. Longer follow-up periods would be necessary to evaluate whether the observed improvements are sustained over time. Third, the study did not objectively assess the students’ ability to perform CPR in a real-life scenario. Finally, the fact that participation was voluntary may have introduced a selection bias, as students who were more motivated or interested in health-related topics might have been more likely to participate, potentially inflating the observed effects of the intervention.

Despite these limitations, the results suggest that school-based CPR training can be feasibly implemented in public school networks, with potential for adaptation in other settings such as rural or low-resource environments. Integration with national education and emergency policies may further reinforce public health outcomes, but further studies with larger, more diverse samples and longer follow-ups are needed.

## Conclusion

The teaching of CPR in educational institutions is a valuable strategy for improving CPR knowledge retention at 3 months following the intervention. This finding supports the feasibility of integrating similar programs in Brazilian public and private schools. These initiatives reinforce Brazilian government projects and efforts to teach these concepts in schools and are aligned with well-established international practices. However, there is a clear need for development of studies that evaluate whether teaching these concepts translates into improved and effective care for patients with CA.

## Data availability statement

The datasets generated and/or analyzed during the current study are available from the corresponding author upon reasonable request.

## AI assistant disclosure

The authors used generative AI tools, including Perplexity AI (Perplexity AI, Inc.) for language editing and clarity improvements, and Gemini (Google LLC) exclusively to enhance image quality without modifying scientific content or underlying data. All manuscript contents, analyses, and conclusions were conceived, verified, and approved by the authors, who take full responsibility for the accuracy and integrity of the work.

## Conflicts of interest

The authors declare no conflicts of interest.

## Appendix Supplementary materials

Image, application 1

## Footnotes

Supplementary material associated with this article can be found in the online version at doi:10.1016/j.bjane.2026.844787.

## References

[1] Kleinman M.E., Buick J.E., Huber N. (2025). Part 7: adult basic life support: 2025 American Heart Association Guidelines for Cardiopulmonary Resuscitation and Emergency Cardiovascular Care. Circulation.

[2] Nakagawa N.K., Carvalho C.R.R., Vieira R.P. (2019). Kids Save Lives Brazil: a successful pilot program to implement CPR at primary and high schools in Brazil resulting in a state law for a training CPR week. Resuscitation.

[3] Semeraro F., Scapigliati A., Ristagno G. (2020). Renewed Kids Save Lives campaign to further increase awareness and fight sudden cardiac death in the era of COVID-19. Resuscitation.

[4] Calderaro M., Navarro J.C., de Carvalho M.R. (2022). The lack of knowledge on acute stroke in Brazil: a cross-sectional study with children, adolescents, and adults from public schools. Clinics (Sao Paulo).

[5] Bottiger B.W., Semeraro F., Wingen S. (2017). Kids Save Lives: Educating schoolchildren in cardiopulmonary resuscitation is a civic duty that needs support for implementation. J Am Heart Assoc.

[6] Schroeder D.C., Ecker H., Wingen S., Semeraro F., Bottiger BW. (2017). Kids Save Lives ‒ resuscitation training for schoolchildren: Systematic review. Anaesthesist.

[7] Semeraro F. (2018). Kids Save Lives ‒ Three years of implementation in Europe. Resuscitation.

[8] Bottiger B.W., Van Aken H. (2015). Kids save lives ‒ Training school children in cardiopulmonary resuscitation worldwide is now endorsed by the World Health Organization (WHO). Resuscitation.

[9] Ribeiro L.G., Germano R., Menezes P.L., Schmidt A. (2013). Pazin-Filho A. Estudantes de medicina ensinam ressuscitação cardiopulmonar a alunos do fundamental. Arq Bras Cardiol.

[10] Kids Save Lives Brasil. Kids Save Lives Brasil – USP [Internet]. São Paulo: Universidade de São Paulo; [citado em 2025 nov 20]. Disponível em: https://sites.usp.br/kidssavelivesbrasil/.

[11] Fernandes J.M.G., Leite A.L.S., Auto B.S.D. (2014). Ensino de suporte básico de vida para alunos de escolas pública e privada do ensino médio. Arq Bras Cardiol.

[12] Zaith P.T.I., Silva S.L., Rocha V.M., Falarino C.S., Francischetti I. (2025). Primeiros socorros para alunos do ensino médio: uma ação educativa em saúde. Saberes Plur.

[13] Chaves A.F.L., Bezerra I.M.P., Rocha R.S. (2018). Reanimação cardiopulmonar nas escolas: avaliação de estratégia educativa. Rev Expressao Catolica Saude.

[14] Soar J., Monsieurs K.G., Ballance J.H. (2010). European Resuscitation Council Guidelines for Resuscitation 2010 Section 9. Principles of education in resuscitation. Resuscitation..

[15] Berden H.J., Hendrick J.M., Willems F.F., Pijls N.H., Knape JT. (1993). How frequently should basic cardiopulmonary resuscitation training be repeated to maintain adequate skills?. BMJ.

[16] Woollard M., Whitfield R., Newcombe R.G., Colquhoun M., Vetter N., Chamberlain D. (2006). Optimal refresher training intervals for AED and CPR skills: a randomised controlled trial. Resuscitation.

[17] Riegel B., Nafziger S.D., McBurnie M.A. (2006). How well are cardiopulmonary resuscitation and automated external defibrillator skills retained over time? Results from the Public Access Defibrillation (PAD) Trial. Acad Emerg Med.

[18] Breckwoldt J., Beetz D., Schnitzer L., Waskow C., Arntz H.R., Weimann J. (2007). Medical students teaching basic life support to school children as a required element of medical education: a randomised controlled study comparing three different approaches to fifth year medical training in emergency medicine. Resuscitation.

[19] Ribeiro L.G., Germano R., Menezes P.L., Schmidt A., Pazin-Filho A. (2013). Medical students teaching cardiopulmonary resuscitation to middle school Brazilian students. Arq Bras Cardiol.

[20] Bohn A., Lukas R.P., Breckwoldt J., Bottiger B.W., Van Aken H. (2015). Kids save lives: Why schoolchildren should train in cardiopulmonary resuscitation. Curr Opin Crit Care.

[21] Connolly M., Toner P., Connolly D., McCluskey DR. (2007). The 'ABC for life' programme: Teaching basic life support in schools. Resuscitation.

