# Supplementary material for: School-based cardiopulmonary resuscitation training for high school students in Brazil: a pre-post educational intervention with 3-month follow-up
Source: Braz J Anesthesiol. 2026 Jul 1;76(5):844787. doi: 10.1016/j.bjane.2026.844787 (PMC13382242; doi:10.1016/j.bjane.2026.844787)
Supplement: Supplementary file 1 [file mmc1.docx]

**BJAN-D-25-00543_Supplementary Material**

- **Questionnaire regarding Expectations over BLS Course.**

1. I am motivated about the Cardiopulmonary Resuscitation course.
2. My current knowledge is sufficient to follow the Cardiopulmonary Resuscitation course.
3. I find it easy to manage a cardiac arrest following the Basic Life Support protocol.
4. In this course, I expect to increase my knowledge about Cardiopulmonary Resuscitation.
5. I expect to find it easy to perform the resuscitation maneuvers.
6. I believe I can satisfactorily learn the content taught in the course.
7. Performing simulation with a manikin during the practical activity will be essential to consolidate the CPR content.
8. I believe that the resources and activities offered in the course will be sufficient for my learning.
9. I expect the course tutors to provide the necessary support for my good performance in the course.
10. I expect the course instructors to be accessible to clarify any questions I may have.
11. I expect this course to provide the necessary information for my learning.
12. I believe that the way the instructor presents the content will be adequate for my learning.
13. I believe that the pre-test applied in this course will be important to guide my attention during the theoretical class.

- **Theoretical questionnaire regarding BLS.**

1. What is the first action to take when a victim suddenly faints?
2. It is 7:39 PM, and you are doing your daily physical activity along the Atalaia waterfront when you come across an individual lying on the ground seven meters in front of you. Among the listed support actions, which should you perform first?
3. After a victim suddenly faints in front of you, how do you check if they are unconscious?
4. How do you check if the victim is breathing?
5. Which of the following victims are in Cardiac Arrest (CA)?
6. What is the emergency number for SAMU?
7. What does AED stand for?
8. Which of the following locations is most likely to have an AED available?
9. You are in a crowded area and a person suddenly collapses in front of you. You call out to them and they do not respond. After confirming the scene is safe, what should you do first?
10. You are performing CPR on a patient in cardiac arrest with effective chest compressions, and at a certain point, the AED arrives. What should you do?
11. Where should chest compressions be performed?
12. What is the ideal rate for performing Cardiopulmonary Resuscitation (CPR)?
13. Chest compressions become effective when the chest compresses by:
14. What is the correct technique for performing chest compressions?

- **Questionnaire regarding Evaluation and Satisfaction over BLS Course**

1. My motivation regarding the topic of Cardiopulmonary Resuscitation increased after this course.
2. My knowledge of Cardiopulmonary Resuscitation was sufficient to follow the course.
3. I found it easy to perform chest compression maneuvers after the practical class.
4. This course increased my knowledge about Cardiopulmonary Resuscitation.
5. I found it easy to follow the step-by-step procedure to manage a cardiac arrest after the theoretical and practical classes.
6. I believe I satisfactorily learned the content taught in the course.
7. Using the manikin during the practical activity was important to consolidate the theoretical knowledge.
8. The course tutors provided the necessary support for my good performance in the course.
9. The course instructor was accessible to clarify my doubts.
10. The course provided me with the necessary information about Cardiopulmonary Resuscitation in Basic Life Support.
11. The instructor’s method of presenting the content was appropriate for my learning.
12. The pre-test conducted in this course was important for me to pay more attention during the theoretical class.
13. I would recommend this course to a friend or a family member.
14. The course is important for society in general.
15. The course should be mandatory in all schools.
